# Latest combination therapies in psoriasis: Narrative review of the literature

**DOI:** 10.1111/dth.15759

**Published:** 2022-08-26

**Authors:** Federico Diotallevi, Matteo Paolinelli, Giulia Radi, Annamaria Offidani

**Affiliations:** ^1^ Dermatological Clinic, Department of Clinical and Molecular Sciences Polytechnic University of the Marche Region Ancona Italy

**Keywords:** biological drugs, combination therapies, psoriasis

## Abstract

Biological therapies revolutionized the treatment of many chronic inflammatory skin diseases, first of all psoriasis, thanks to their high efficacy and the reduced number of side effects. However, the use of a single biologic drug does not always provide complete control of the disease or associated comorbidities over time. The first biological drugs used for the treatment of psoriasis, tumor necrosis factor alpha inhibitors, have long been used in combination with traditional topical and systemic therapies to induce a complete remission of the disease that could not be achieved with innovative drug alone. Even with the advent of new biological therapies with more precise molecular targets, the challenge of using combination therapies remained. Psoriatic patients often have major comorbidities, such as arthritis, inflammatory bowel disease, uveitis or have other concomitant conditions such as chronic spontaneous urticaria and atopic dermatitis, which may require different biologic treatments than those indicated in psoriasis. The objective of this article is, through a comprehensive revision of the literature, to analyze in which cases the use of the combination of the latest therapies for psoriasis may be useful.

## 1 INTRODUCTION

Biological drugs and in particular modern interleukin (IL) inhibitors revolutionized the treatment of plaque psoriasis in terms of high efficacy and safety.^1^ However, monotherapy is not always sufficient for the global control of the disease, for example in case of episodes of relapse of the disease beyond the therapy, or in case of concomitant psoriatic arthritis (PsA), or in case of involvement of sensitive areas such as scalp, inverse areas, palmoplantar or genital, or in case of concomitant atopy.^2^

Regarding traditional therapies, the combination of different types of topical therapies or traditional systemic therapies is widespread. For example, as a combination of two topical therapies, the use of steroids along with steroid‐sparing agents such as calcipotriene and tazarotene is diffuse. These drugs used separately have less efficacy than steroids but used together can maximize efficacy with fewer side effects.^3^, ^4^

Phototherapy, including ultraviolet B (UVB) and psoralen plus ultraviolet A, is also often used for patients with psoriasis not controlled by topical therapies alone. It is used in combination with the same topical therapies such as vitamin D, vitamin A derivatives, or coal tar, or in combination with systemic therapies such as acitretin, methotrexate, or apremilast.^5^, ^6^, ^7^, ^8^ This combination is used, for example, in cases of psoriasis of difficult areas, such as palmoplantar psoriasis in which acitretin and phototherapy have been shown to be more effective than single treatments.^6^, ^7^ While the combination of two conventional systemic treatments, such as acitretin and methotrexate, is usually not recommended due to the increased number of side effects, the combination of phototherapy or conventional systemic therapies with biologic therapies is often performed. Tumor necrosis factor alpha (TNF‐alpha) inhibitors adalimuamb and etanercept and IL 12/23 inhibitor, have been shown to achieve higher skin clearance in less time when used in combination with nb‐UVb phototherapy.^9^, ^10^, ^11^, ^12^ In addition, the combination of etanercept, adalimumab, and infliximab with methotrexate is widely used especially in cases of concomitant psoriasis and PsA for a better control of arthritic symptoms.^13^, ^14^, ^15^

However, much less evidence exists on the combination of two biologic drugs or the combination of biologic drugs and small molecules.

The aim of this review is to analyze in which cases these combinations have been used and in which cases they can be effective.

## 2 MATERIALS AND METHODS

This narrative review was based on the general approach developed biomedical narrative review construction, that involves four key steps: 1‐identify keywords; 2‐conduct research; 3‐review abstract and articles; 4‐document results.^16^

### 2.1 Identify keywords

To identify keywords, a brainstorming approach, involving the entire research group, was used. The research team consisted of four dermatologists experienced in psoriasis pathophysiology. Two among them had also specific expertise in psoriasis treatment, and one in literature review methodology.

During the first meeting, the research team selected the topic, identified the scope, constructed the title, and selected the keywords as follows: “combination therapy AND psoriasis”, “psoriasis AND combination of biological therapy”, “combination AND apremilast AND biologics AND psoriasis”, “combination AND dupilumab AND biologics AND psoriasis”, “combination AND xolair AND biologic AND psoriasis”.

### 2.2 Conduct research

An extensive search for eligible articles was conducted on the following databases: National Library of Medicine PubMed database, and Scopus. The references list of selected studies was scanned to find additional records.

Inclusion criteria: studies reporting on psoriasis as immune‐mediated disease, published in English language, published between 1990–2020, abstract available. No restriction on the design of the study was considered, and randomized controlled trial, case–control study, cross‐sectional study, case reports and series, and review article were included.

### 2.3 Review abstract and title

The selection of the relevant data published in the literature took place in three steps. In the first step, two researchers (F.D., G.R.) independently selected the articles based on the title. Any disagreement was solved by consulting a senior investigator (A.O.). The second step consisted of evaluating the abstracts. At least two members of the research team (M.P. and G.R.) independently assessed each abstract. The research team resolved all discrepancies through consensus. Linguistic revision was performed by A.O.

### 2.4 Document results

All sources with similar data/level of evidence were analyzed, collected, and grouped. The main text was structured into subsections. New evidence‐based points were summarized and major points for future research and practice were defined.

## 3 RESULTS

Two macro topics emerged from the literature review: the combined use of biologic drugs in patients with plaque psoriasis and the combined use of biologic drugs and small molecules in plaque psoriasis.

### 3.1 Association of two biological drugs

Biological drugs allow, through the blockade of one or few molecules, to selectively inhibit a specific biological pathway with greater efficacy and usually less adverse events than traditional immunosuppressants. However, the pathogenesis of chronic immune‐mediated inflammatory diseases and in particular of psoriasis, depends on the role of multiple inflammatory pathways, often closely interconnected. It is not easy to understand which one could prevail in different subsets of patients, even if they suffer from the same disease from a clinical point of view.^17^ For this reason, it is not always possible to achieve optimal disease control using only one biological drug.

In a complex disorder such as psoriasis where several organs may be involved, especially skin and joints, the use of a single biological drug may not be optimal for all aspects of the disease or even paradoxically aggravate some symptoms. For example, antiTNF‐α drugs that generally have good efficacy in the treatment of PsA can sometimes be less effective on the skin component or even promote its exacerbation with the so‐called paradoxical psoriasis phenomenon.^17^, ^18^, ^19^, ^20^ Moreover, psoriatic patients often present important comorbidities, such as inflammatory bowel diseases (IBD) or uveitis, or have other concomitant conditions such as chronic spontaneous urticaria (CSU) and atopic dermatitis (AD), which may require different biological treatments than those indicated in psoriasis. While in other branches of medicine, the combination of biological drugs is frequent and well established, in dermatology the association of biological treatments is rarely described mainly due to safety concerns.^21^, ^22^ Since there are no precise guidelines or biomarkers to orient the prescription of a dual biological therapy, most of our knowledge derives from case reports.

#### 3.1.1 Combination of biological treatments for the control of recalcitrant psoriatic disease

Usually, switching from one class of biologicals to another is the most common practice adopted by clinicians in case of inefficacy of one drug. However, psoriatic patients who have failed two or more biologic therapies are not uncommon in everyday clinical practice.^23^ Switching to a third drug, or even more, is not always easy because the patient may have comorbidities that limit the use of some class of biologics, conventional DMARDs or small molecules. For example, in patients with IBD, frequently associated with psoriasis, the use of IL‐17A inhibitors is usually considered improper.^24^ In addition, conventional DMARDs and small molecules have broad‐spectrum immunomodulatory mechanisms and drug interactions that may limit their use in elderly, frail patients or those with other comorbidities. In these selected cases, the dermatologist may consider a dual biologic therapy, especially combining agents that target different inflammatory pathways. In the current scenario are available antipsoriatic biological drugs that antagonize TNF‐α, IL‐17 and IL‐23 (with ustekinumab targeting both IL23 and IL‐12). In gastroenterology, combinations of two biologics are already used primarily for three reasons:

1) To synergistically compensate the loss of efficacy of an ongoing biologic therapy (rescue therapy);

2) To counteract the adverse events of a biological therapy (i.e., addition of ustekinumab to a TNF inhibitor to counteract a paradoxical psoriasis caused by the latter);

3) For the rapid control of very severe cases in which a fast‐acting drug can act as an initial bridge to a second, slower‐acting drug that could then be used as monotherapy.^25^

These situations can be encountered also in the dermatological clinical practice and there are an increasing number of reports of biological drugs combinations for the management of challenging cases. The association of biological drugs often allows to achieve the control of the disease, cutaneous and/or articular, in multifailure patients or in those who have already tried the combination of biologics with conventional DMARDs or other oral systemic agents. Despite safety concerns in combining two biological drugs, few reports of serious side effects emerge from the current literature (Table 1).^26^, ^27^, ^28^, ^29^, ^30^, ^31^, ^32^ When considering a dual biological therapy, it is mandatory to weigh all risk and benefits for the patient and collaborate with other specialists in choosing the association for the management of the comorbidity.

**TABLE 1:** Reported cases of dual biologic therapy for psoriasis and psoriatic arthritis.

Patient Age/sex (ref)	Diagnosis	Prior therapies	Dual biologic therapy	Dose	F.U. (months)	Adverse effects
62/M^26^	PsO + PsA	UST, IFX, ADA, ETN	UST + ETN	90 mg Q4W + 50 mg QW	6	Unstable angina
33/M^27^	Palmoplantar pustulosis	Mycophenolate, ADA	UST + ADA	90 mg Q12W + 40 mg QW	8	None
67/M^28^	PsO + PsA	IFX, ETN, ADA, UST	UST + ETN	90 mg Q12W + 50 mg QW	62	Herpes zoster flares
39/M^26^	PsO + PsA	ETN, ADA	UST + ETN	90 mg Q12W + 25 mg QW	50	Retrotonsillar abscess
49/F^26^	PsO + PsA	ETN, EFA, IFX, ADA, UST	UST + ADA UST + GOL	90 mg Q12W + 40 mg QoW 90 mg Q12W + 100 mg Q4W	25 7	Erysipelas, pneumonia Erysipelas
44/F^26^	PsO + PsA	ETN, IFX, ADA	UST + ADA UST + CZP	90 mg Q12W + 40 mg QoW 90 mg Q12W + 200 mg QoW	18 36	None None
38/M^29^	PsO + PsA	IFX, ADA, ETN, ABA, UST	UST + ETN	90 mg Q4W + 50 mg QW	>12	None
23/F^29^	PsO + PsA	UST	UST + ETN UST + ADA	90 mg Q8W + 25 mg BiW 90 mg Q8W + 40 mg QW	8 N.R.	None Foruncles, autoimmune hemolytic anemia
46/F^30^	PsO + PsA	ADA, GUS	GUS + ADA	100 mg Q8W + 40 mg QoW	6	None
38/F^31^	PsO + PsA	ADA, IFX, ETN, GOL, UST, SEC, GUS	UST + ETN SEC + ETN GUS + ETN	45 mg Q12W + 50 mg QW 300 mg QoW +50 mg Q4W 100 mg Q4W + 50 mg QW	12 6 15	↑ UTI; severe H2N1 flu None None
NA M^32^	PsO + PsA	Mtx	RKZ + GOL	150 mg Q12W + 50 mg Q4W	12	None

Abbreviations: ABA, abatacept; ADA, adalimumab; BiW, twice weekly; CZP, certolizumab pegol; EFA, efalizumab; ETN, etanercept; F, female; GOL, golimumab; GUS, guselkumab; IFX, infliximab; M, male; PsA, psoriatic arthritis; PsO, psoriasis; Q4W, every 4 weeks; Q12W, every 12 weeks; QoW, every other week; QW, once a week; RKZ, risankizumab; SEC, secukinumab; SSz, sulfasalazine; UST, ustekinumab; UTI, urinary tract infections.

#### 3.1.2 Combination of biological treatments for concomitant different immune‐mediated diseases

Multiple immune‐mediated diseases are often associated in the same patient. It is not always possible to obtain a satisfactory remission of different diseases with a single immunomodulatory therapy due to very distinct pathogenetic mechanisms. For this reason, the use of more than one biological drug in the same patient with multiple autoimmune diseases is described in the literature. As mentioned in the previous chapter, the combination of an antipsoriatic biological drug and a biological drug targeted for chronic IBD is already performed in gastroenterology. In addition Diluvio et al. described the case of an adult female affected by psoriasis vulgaris and CSU who received treatment with respectively tildrakizumab and omalizumab to obtain the remission of both diseases.^33^ The same author reported the case of a young female patient who developed CSU resistant to antihistamines during the treatment with adalimumab for PsA. The co‐administration of omalizumab for 24 weeks was successful in inducing the remission of CSU without any significant adverse event. In addition, 6 months after the discontinuation of the anti‐IgE biological drug the patient remained urticaria free.^34^

Few cases in the literature are reported of association between biologics for psoriasis (PsO) and AD. However, the clinical overlap of psoriasis and Th2‐mediated diseases is increasingly common. In these patients, the two Th17 versus Th2 pathways apparently at the antipodes on the molecular profile, seem to present a range of intermediate phenotypes for which, at the moment, each single disease requires specific treatment.

A retrospective review by Barry et al. reported the data of seven patients with PsO in biological therapy with guselkumab, treated simultaneously with dupilumab for the coexistence of Th2‐mediated diseases.^35^ The authors conducted an electronic registry search of patients treated with dupilumab and an Food and Drug Administration approved psoriasis biologic, belonging to The Tufts Medical Center Department of Dermatology from January 1st, 2016 to May 1st, 2019, with a minimum follow‐up of 6 weeks from initiation of combined therapy. Seven patients met the criteria: all patients were affected by psoriasis and six of them suffered from AD and one from bullous pemphigoid (BP). All seven patients have completed combination dupilumab/guselkumab treatment for 2–13 months (mean = 6.4, median = 6). All six AD patients experienced significant clinical improvement after dupilumab therapy and five of six experienced a satisfactory clinical psoriasis response to guselkumab. Only one patient improved AD but not PSO. The patient with PsO and BP also responded to the treatments: the bullous lesions resolved without new ones developing; the PsO also improved.

Regarding safety, only mild injection site reaction was observed in one patient. The small sample, the lack of long‐term observation and the retrospective nature referred to a single center are the main limitations of the study.

### 3.2 Association of biological drugs and small molecules

Target therapy represented a turning point in the treatment of psoriasis. However, not all patients respond adequately to the various biological treatments: some lose efficacy and others are multifailure to multiple therapies. Therefore, combined therapies are useful in some cases. Apremilast is an oral small molecule, phosphodiesterase four inhibitor indicated for the treatment of PsO and PsA. We report data from the literature in which apremilast has been used in combination with biological drugs for the treatment of psoriasis.

Abu Hila et al. conducted a retrospective study to evaluate the short‐term efficacy and safety of apremilast in combination with at least one other systemic therapy for chronic plaque psoriasis. Among the subjects in treatment with biologic drugs (*n* = 32) who received apremilast, 13 patients were treated with TNF‐alpha inhibitors (7 infliximab, 4 etanercept, 2 adalimumab), 6 patients received both methotrexate and 1 TNF‐alpha inhibitor (5 adalimumab and methotrexate, 2 infliximab, and methotrexate) and 13 patients were on ustekinumab.^36^ All patients received apremilast for 12 weeks in combination with ongoing biological therapy. A total of 26 of 32 patients (82%) achieved PASI 75 after 12 weeks of combined therapy and in detail 10 of 13 (77%) in the apremilast + TNF‐alpha inhibitors group, 5 of 6 (83%) in the apremilast + methotrexate and TNF‐alpha inhibitors group, 11 of 13 (85%) in the apremilast + ustekinumab patient group. Among 32 patients 9 experimented nausea and or diarrhea (about one third for each group) and 5 referred weight loss.

Several case reports and case series are reported on the association between apremilast and secukinumab to treat recalcitrant psoriasis. Sacchelli et al described a case series of four patients with psoriasis refractory to previous traditional systemic treatments (phototherapy, methotrexate [MTX], ciclosporin A, acitretin) and to all the available biologic therapies BTs (infliximab, adalimumab, etanercept, and ustekinumab).^37^ Patients received combination therapy (CT) with secukinumab and apremilast for a variable period of 6–9 months. Three of four patients achieved PASI 75 and one patient achieved PASI 100. No serious adverse events were noted, other than mild diarrhea coinciding with the beginning of the treatment with apremilast, and that became self‐limited over time. The authors considered the CT with apremilast a valid and safe alternative to other traditional systemic drugs to the association with biological drugs.

Rothstein et al. described a case report of a 67‐year‐old Caucasian man receiving secukinumab with progressive loss of clinical response 6 months after starting treatment. The addition of apremilast induced improvement of psoriasis after 3 months, with minimal residual lesions (BSA 5%) and initial mild diarrhea as an adverse event.^38^

Nisal et al. described the case of a 23‐year‐old patient with recalcitrant PsO and PsA. He had failed previous treatments with cyclosporine (discontinued for severe mood changes), sulfasalazine (discontinued for erythrodermal flare‐up), methotrexate, adalimumab monotherapy for 3 years, which was discontinued following secondary failure and ustekinumab, discontinued after 9 months for primary ineffectiveness. He started apremilast therapy with slight improvement in joint side at 12 weeks, but not in skin counterpart.^39^ For this, secukinumab was added and after 16 weeks he obtained an absolute clearance of the skin and a further improvement in PsA. A total of 9 months later, the disease remained well controlled with no reported adverse events.

More information on safety is reported by Metyas et al.^40^ The authors conducted a retrospective, open label, single‐center study to determine the safety of apremilast used in combination of biologic therapies in the treatment of PsO and PsA. They enrolled 22 patients under biologic treatment and apremilast was added to their current biologic agent. Out of 22 patients, six patients developed side effects, none of which caused discontinuation of therapy. Out of the six patients who developed side effects, two patients developed nausea and two patients developed diarrhea. One patient developed weight loss and one patient experienced abdominal pain. A summary of the cases can be found in Table 2.

**TABLE 2:** Reported cases of combination of biologic therapy and apremilast in plaque psoriasis.

Type of article (ref)	N of patient	Mean PASI before CT	Mean PASI after CT	Patients achieved PASI 75 (*n*; %)	Duration of CT	AE (*n*)	Biologic drug combined to Apremilast
Retrospective study AbuHila et al^36^	13	8,1	2,4	10; 77%	12 weeks	Nausea and/or diarrhea^3^ Weight loss^2^	TNF Inhibitors
	6	9,9	2,3	5; 83%		Nausea and/or diarrhea^2^ Weight loss^1^	TNF Inhibitors/Methotrexate‐
	13	8,4	1,9	11;85%		Nausea and/or diarrhea^4^ Weight loss^2^	ustekinumab‐
Case series Sacchelli et al^37^	4	8	2	4 (100%)	6–9 months	Nausea and/or diarrhea^4^	secukinumab
Case report Rothstein et al^38^	67‐year‐old Caucasian man	n/a	n/a	yes	12 weeks	Nausea and/or diarrhea	secukinumab
Case report Nisal et al^39^	23‐year‐old patient with PsO and PsA	16,2	0	yes	16 weeks	None	secukinumab
Retrospective study^40^ Metyas et al.^41^	22	n/a	n/a	n/a	n/a	Nausea and/or diarrhea^4^ Weight loss^1^ Abdominal pain^1^	n/a

Abbreviations: AE, adverse events; CT, combination therapy; n/a, not applicable.

## 4 CONCLUSION

Nowadays, thanks to the new knowledge of immuno‐pathology, many advances have been made in the knowledge of psoriatic disease, which is considered a systemic disease in which the skin is only one of the organs involved.^41^, ^42^ It is also a chronic condition with episodic flares, requiring creative treatments to treat both acute flare and maintain long‐term disease control. It is also associated with numerous comorbidities many of which require additional treatment beyond that for psoriasis. From this perspective, taking care of psoriatic patients is not easy, and a holistic, multidisciplinary approach is often necessary.^43^

Biologic therapies for psoriasis have revolutionized the treatment of the condition, and new drugs, especially antiILs, allow optimal control of the disease with low side effects.^1^, ^44^ However, the use of increasingly targeted molecular targets if on the one hand reduces side effects, on the other hand also reduces the therapeutic range of action in case of acute flares or in case of concomitant diseases.

The combination of biologic drugs or the latter with small molecules with different mechanisms of action and side effect profiles, either concomitantly or as part of a rotational or sequential therapy, may be an effective method to achieve disease control in some patients with psoriasis. However, there is no unanimous consensus in the scientific community on the combination of biologic drugs. Certainly, one possible disadvantage is the high cost of the therapies although the use of biosimilars has greatly lowered the price of some biologic drugs.^45^ The authors' intention is not to endorse the indiscriminate use of combination of biologic drugs, but to offer a panoramic of new therapeutic strategies of combination of innovative drugs that are already being combined within other medical disciplines. The hope is that the study of the immunologic profile of immune‐mediated inflammatory diseases, cutaneous and other, will be able to produce drugs with multiple therapeutic targets in the future that can simultaneously treat multiple diseases in patients.

## AUTHOR CONTRIBUTIONS

Federico Diotallevi: concept and design, literature search, drafting of manuscript, revision. Giulia Radi: literature search and drafting of manuscript. Matteo Paolinelli: literature search and drafting of manuscript. Annamaria Offidani: final revision. All named authors meet the International Committee of Medical Journal Editors (ICMJE) criteria for authorship for this article, take responsibility for the integrity of the work as a whole, and have given their approval for this version to be published.

## CONFLICTS OF INTEREST

All authors contributed to the writing of the manuscript. The authors declare no conflicts of interest. Federico Diotallevi, Giulia Radi, Matteo Paolinelli, and Annamaria Offidani have nothing to disclose.

## FUNDING STATEMENT

No funding or sponsorship was received for this study or publication of this article.

## ETHICS STATEMENT

This article is based on previously conducted studies and does not contain any new studies with human participants or animals performed by any of the authors.

## Data Availability

Data sharing is not applicable to this article as no new data were created or analyzed in this study.
